# Simple cystic lesions of the pancreas: image quality and diagnostic accuracy of photon-counting detector computed tomography

**DOI:** 10.1007/s11547-025-02015-w

**Published:** 2025-04-21

**Authors:** Stephan Rau, Thomas Stein, Alexander Rau, Caroline Wilpert, Fabian Pallasch, Balazs Bogner, Sebastian Faby, Fabian Bamberg, Jakob Weiss

**Affiliations:** 1 Department of Diagnostic and Interventional Radiology, Medical Center – University of Freiburg, Faculty of Medicine, University of Freiburg, Hugstetter Str. 55, 79106 Freiburg im Breisgau, Germany; 2 Computed Tomography, Siemens Healthineers AG, Siemensstr. 3, 91301 Forchheim, Germany

**Keywords:** Tomography, X-ray computed, Photon-counting CT, Photon-counting detectors, Pancreatic ductal*/diagnostic imaging, Humans, Pancreas, Pancreatic intraductal neoplasms*/diagnosis, Pancreatic neoplasms*/diagnostic imaging

## Abstract

**Purpose:**

To evaluate image quality and diagnostic accuracy of photon-counting detector (PCD) CT for the detection of pancreatic cystic lesions (PCLs) compared to energy-integrating detector (EID) CT with MRI serving as reference standard.

**Material and methods:**

We included consecutive patients who underwent contrast-enhanced PCD-CT of the abdomen and for whom an additional abdominal EID-CT was available. Multiparametric MRI served as the reference standard. CT images were assessed for the presence of PCLs by three radiologists independently in a blinded reading. Image quality, lesion conspicuity, and diagnostic confidence were rated on a 5-point Likert scale (5 = excellent). The coefficient of variation (CV) and the density difference between PCLs and visually normal pancreatic parenchyma were calculated as quantitative imaging measures. Radiation dose was assessed using CTDIvol [mGy].

**Results:**

Among 106 included patients (age 62.7 ± 12.6 years; 45 [42.5%] male), 46 had MRI-confirmed cystic lesions (mean size 8.7 ± 7.4 mm; range 2–45 mm). Diagnostic accuracy for PCLs was significantly higher for PCD-CT vs. EID-CT (area under the curve: 0.81 vs. 0.74; *p* = 0.002; sensitivity: 76.8% vs. 59.4%). Image quality, lesion conspicuity, and diagnostic confidence were rated superior for PCD-CT vs. EID-CT (all *p* < 0.001). Quantitative analyses revealed a significantly lower CV (0.19 vs. 0.24; *p* = 0.002) and a higher density difference (94.1 HU vs. 76.6 HU *p* < 0.001) between PCLs and visually normal pancreatic parenchyma at lower radiation doses (7.13 vs. 8.68 mGy; *p* < 0.001) for PCD-CT vs. EID-CT.

**Conclusion:**

PCD-CT provided significantly higher diagnostic accuracy and superior image quality for the detection of PCLs compared to conventional EID-CT at lower radiation dose.

## Introduction

Pancreatic cystic lesions (PCLs) are a frequent incidental finding, which can be detected in up to 24–49% of cross-sectional abdominal imaging studies posing significant diagnostic challenges [1, 2]. To this date, the ACR incidental findings committee and the new Kyoto guidelines recommend follow-up imaging for PCLs of any size without clear signs of malignancy because several studies with histopathological workup have demonstrated that apparently simple PCLs in asymptomatic patients comprise a wide range of pathologies including a significant number of premalignant and malignant lesions [3–6]. Consequently, detection of any PCL in abdominal imaging studies is crucial for appropriate and personalized patient management.

MR imaging is considered the reference standard for detailed assessment of PCLs mainly relying on the high contrast in T2-weighted sequences complemented by contrast-enhanced and diffusion-weighted imaging [7]. In CT imaging, the detection of PCLs is limited by their low contrast in comparison with surrounding pancreatic parenchyma and visceral adipose tissue, which explains the substantially lower detection rate compared to MR imaging studies (up to 5% in CT vs. up to 20% in MR) [8]. Yet, given the limited availability, and longer examination times of MRI, increasing CT-based detection of PCLs would be desirable to improve patient management, streamline diagnostic workflows, and optimize healthcare costs. In this context, the recently introduced photon-counting detector (PCD) technology, which facilitates higher spatial resolution and improved image contrast, may help to overcome current limitations of conventional energy-integrating detector (EID) CT systems for PCL detection.

The purpose of this study was to evaluate the image quality and diagnostic accuracy of PCD-CT for the detection of PCLs compared to conventional EID-CT with MRI serving as reference standard.

## Methods

### Case series

In this exploratory cross-sectional case series, we included consecutive patients between February 2021 and October 2023 who underwent clinically indicated contrast-enhanced PCD-CT or EID-CT of the abdomen and required a clinically indicated follow-up imaging. To allow for a direct comparison between PCD-CT and EID-CT, the follow-up scan was scheduled on the CT system that was not used to acquire the baseline scan. In addition, a multiparametric abdominal MRI was arranged for these patients during the same time frame, if not already available and if clinically indicated. Patients were excluded if a major surgical procedure on the pancreas was performed in between the three examinations. Additionally, individuals under 18 years of age were excluded.

The local Institutional Review Board (Ethics Committee of the University Medical Center Freiburg, case number 21-2469) approved this prospective study, and written informed consent was obtained from all patients prior to study inclusion.

### CT Image acquisition and reconstruction

#### PCD-CT

All PCD-CT scans were acquired on a first-generation dual-source PCD-CT scanner (NAEOTOM Alpha, Siemens Healthineers, Forchheim, Germany). Patients underwent body weight-adapted (1.2 mL/kg of below-mentioned contrast agent) contrast-enhanced CT imaging of the abdomen in the portal venous phase with a fixed bolus delay of 80 s after contrast agent administration. Contrast agent (Iopromide, Ultravist 370 mg iodine/mL, Bayer Healthcare, Leverkusen, Germany) was administered using a dual-syringe power injector (Accutron CT-D Vision, Medtron, Saarbrücken, Germany) with a flow rate of 4.0 mL/s followed by a saline flush (40 mL; flow rate of 4.0 mL/s).

Acquisition parameters were as follows: tube voltage 120 kVp, automated attenuation-based tube current modulation with an image quality level (IQ Level) of 145, pitch factor 0.8, collimation 144 × 0.40 mm, and 0.5 s rotation time.

From the acquired data, two series of virtual monoenergetic images at 65 keV were reconstructed in axial orientation using a standard soft tissue kernel (Br40) and Quantum Iterative Reconstruction strength 4: (1) standard series with a slice thickness of 3.0 mm and an increment in 3 mm; (2) thin sections with a slice thickness of 1.0 mm and an increment of 1 mm. Virtual monoenergetic reconstructions at 65 keV were chosen based on preclinical and clinical studies which have demonstrated optimal CNR at settings between 60 and 70 keV [9, 10].

#### EID-CT

All EID-CT scans were performed on a third-generation dual-energy EID-CT scanner (SOMATOM Force, Siemens Healthineers, Forchheim, Germany). Portal venous phase images were acquired using the same contrast agent protocol as detailed above.

Acquisition parameters were as follows: dual-energy mode with a tube voltage combination of 90 kV and Sn150 kV, automated attenuation-based tube current modulation with quality reference mAs of 152 mAs and 95 mAs for tube A and B, respectively, 0.5 s rotation time, pitch factor 0.6, and collimation 128 × 0.6 mm.

Similar to the PCD-CT scans, two series in axial orientation (3-mm and 1-mm slice thickness and increment, respectively) were reconstructed from the acquired data using the 90 keV scans, a standard soft tissue kernel (Bf40) and Advanced Modeled Iterative Reconstruction (ADMIRE) with strength 3. The mean energy level of the 90 kV scan lies between 60 and 65 keV which allows a reasonable comparison to the monoenergetic reconstructions from the PCD-CT [11].

### Diagnostic accuracy for the detection of PCL

Diagnostic accuracy was independently assessed by three radiologists with 2, 3 and 7 years of experience in CT imaging. The presence of any PCLs (regardless of size or presence of additional imaging features) was evaluated on a dedicated workstation using a binary (yes/no) rating with the readers blinded toward the CT system, the indication for the scan, and the clinical diagnosis. Both the 3-mm and 1-mm axial reconstructions were provided for review. Additional multiplanar reformations could be reconstructed on demand by the raters within the reading software for verification of findings. If multiple lesions were present, only the largest PCL was assessed to ensure consistency between the readers and to focus on the lesions with most clinical relevance. Readers should also indicate whether worrisome or high-risk features are present if a PCL is detected (e.g., main duct dilations, solid components, or septations), using a binary rating (yes/no).

The reference standard to determine if a PCL was present was based on the multiparametric MRI study including T2-weighted sequences in axial and coronal orientation, contrast-enhanced series, and diffusion-weighted imaging. To generate the best possible reference, a dedicated study read was performed by three radiologists in consensus with 4, 6 and 10 years of experience in MR imaging, who did not participate in the CT reading. The consensus reading also assessed the maximum lesion diameter and appearance of worrisome or high-risk features.

### Qualitative image analysis

Besides assessing the presence/absence of PCL, the same three radiologists participating in the CT reading session evaluated the CT images with respect to (1) overall image quality, (2) lesion conspicuity, if present, and (3) diagnostic confidence in a similar fashion as outlined above. All readings used a 5-point Likert scale (1 = non-diagnostic, 2 = poor image quality and lesion conspicuity/low confidence, 3 = moderate image quality and lesion conspicuity/moderate confidence, 4 = good image quality and lesion conspicuity/good confidence, and 5 = excellent image quality and lesion conspicuity/excellent).

### Quantitative image analysis

#### Quantitative assessment of lesion conspicuity

For PCLs larger than 5 mm in diameter, the difference in Hounsfield units (HU) between the lesion and the surrounding visually normal pancreatic parenchyma was evaluated as a quantitative measure of lesion conspicuity. First, a region of interest with a fixed area of 80 mm^2^ was drawn in visually normal pancreatic tissue in the 3 mm reconstruction in axial orientation (by SR) intentionally avoiding vessels, ducts, calcifications and visceral adipose tissue. Second, to measure the HU values of the PCLs, a line profile was drawn to have a less noise-dependent evaluation of attenuation within the lesions. Especially for small lesions, line profiles provide the advantage of better delineation of lesion edges compared to ROI measurements, thus reducing the influence of partial volume effects on the absorption values within the cyst. In the line profiles, the mean HU value of the PCL was measured as mean values of all measurement points within the plateau of the line profile, whereby the borders of the plateau were defined via a cutoff attenuation increase of more than 10%. The delta HU between the PCL and visually normal pancreatic parenchyma was assessed using the following formula:

1 $${\varvec{\Delta}}\mathbf{H}\mathbf{U}= {\text{HU}[\text{ROI}}_{\text{PP}}]-\text{ HU}[{\text{LP}}_{\text{PCL}}]$$

with pp = pancreas parenchyma and LP = line profile.

**Fig. 1:**
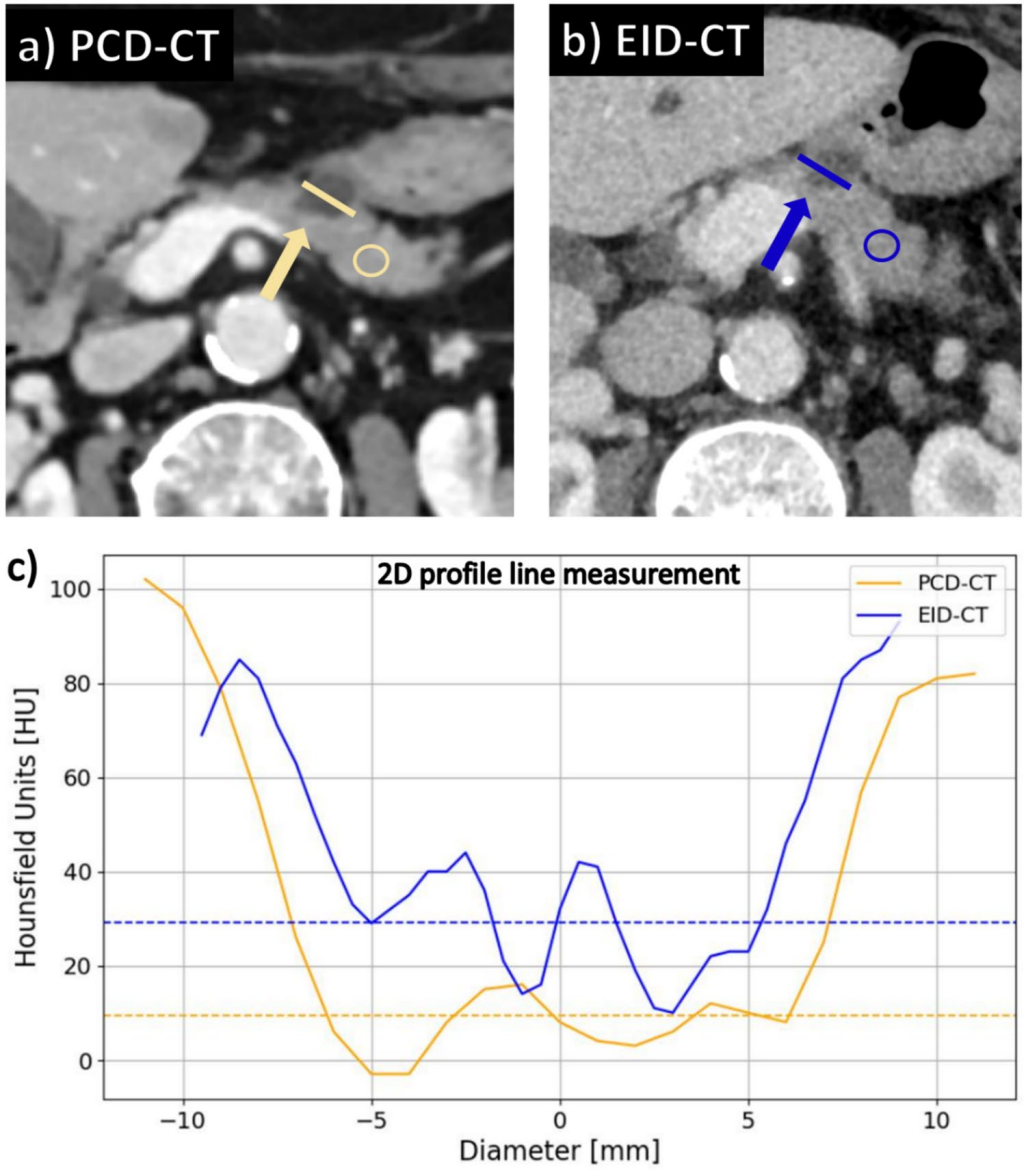
Assessment of quantitative lesion conspicuity. Axial reconstructions from PCD-CT (**a**) and EID-CT (**b**) at the level of the pancreatic corpus showing a cystic lesion measuring 10 × 11 mm (yellow and blue arrow). The mean HU values of normal pancreatic tissue were determined using a region of interest measurement (yellow and blue circle). The diagram (**c**) shows HU measurement of the pancreatic cystic lesion using a 2D line profile for the PCD-CT (yellow line) and EID-CT (blue line) indicating, on average, lower HU values within the cyst for PCD-CT vs. EID-CT with 9.4 HU vs. 29.12 HU (yellow and blue dashed lines). PCD-CT = photon-counting detector computed tomography; EID-CT = energy-integrating detector computed tomography; HU = Hounsfield units

An image example is provided in Fig. 1.

### Evaluation of CT signal homogeneity

To obtain a quantitative measure of the homogeneity of the acquired CT signal independent of the absolute HU values as baseline HU values might vary due to calibration, scanner technology, or image reconstruction algorithms. The coefficient of variation (CV) was calculated in a reference region with the low inter-individual density deviations. Therefore, a ROI was placed in the left paramedian autochthonous muscles intentionally avoiding intermuscular adipose tissue and vasculature. ROI size (150 mm^2^) and location were kept similar in PCD-CT and EID-CT scans to ensure a reliable comparison. The CV is calculated using the following equation:

2 $$\text{CV}=\frac{{\sigma }_{\text{ROI}}}{{\mu }_{\text{ROI}}}$$

where *σ* represents the standard deviation and *μ* the mean HU in the ROI.

### Radiation dose

The radiation dose was assessed and compared between PCD-CT and EID-CT scans for patients examined in routine supine position using the CTDI_vol_.

### Statistical analysis

Continuous variables are given as mean ± standard deviation (SD) or as median and interquartile range (IQR). Categorical variables are expressed as frequencies and percentages. Baseline demographics were compared using the Student’s t test or the Wilcoxon test for continuous variables, depending on the data distribution. For categorical variables, the Chi-square test was used.

To assess the diagnostic accuracy of PCD-CT vs. EID-CT for the detection of PCL, receiver operating characteristic (ROC) curves and the area under the curve (AUC) were calculated and compared using the Delong test. In addition, sensitivity and specificity were calculated for all PCLs, both pooled for all readers and for each reader individually. Sensitivity analyses were performed for PCLs with a threshold diameter for lesions ≥ 5 mm and lesions ≥ 10 mm. Size measurements of the MRI-verified PCLs were compared between MRI, PCD-CT, and EID-CT including absolute differences of measurements in mm. Cases where PCLs were not identified in the reading were excluded to ensure reliable comparisons of size measurements across modalities. Additional sub-analyses of lesions larger ≥ 5 mm and lesions ≥ 10 mm were performed.

Reading scores of the qualitative image analysis were pooled for all readers and compared using the Wilcoxon signed rank test. For all quantitative image analyses and radiation dose assessments, paired t tests were conducted. Size measurements between MRI and the CT systems were compared via repeated measure ANOVA and post hoc pairwise comparison using the Tukey method. The alpha level was set to 0.05. All statistical analyses were performed using Python (version 3.10.12).

## Results

### Patients characteristics

**Fig. 2:**
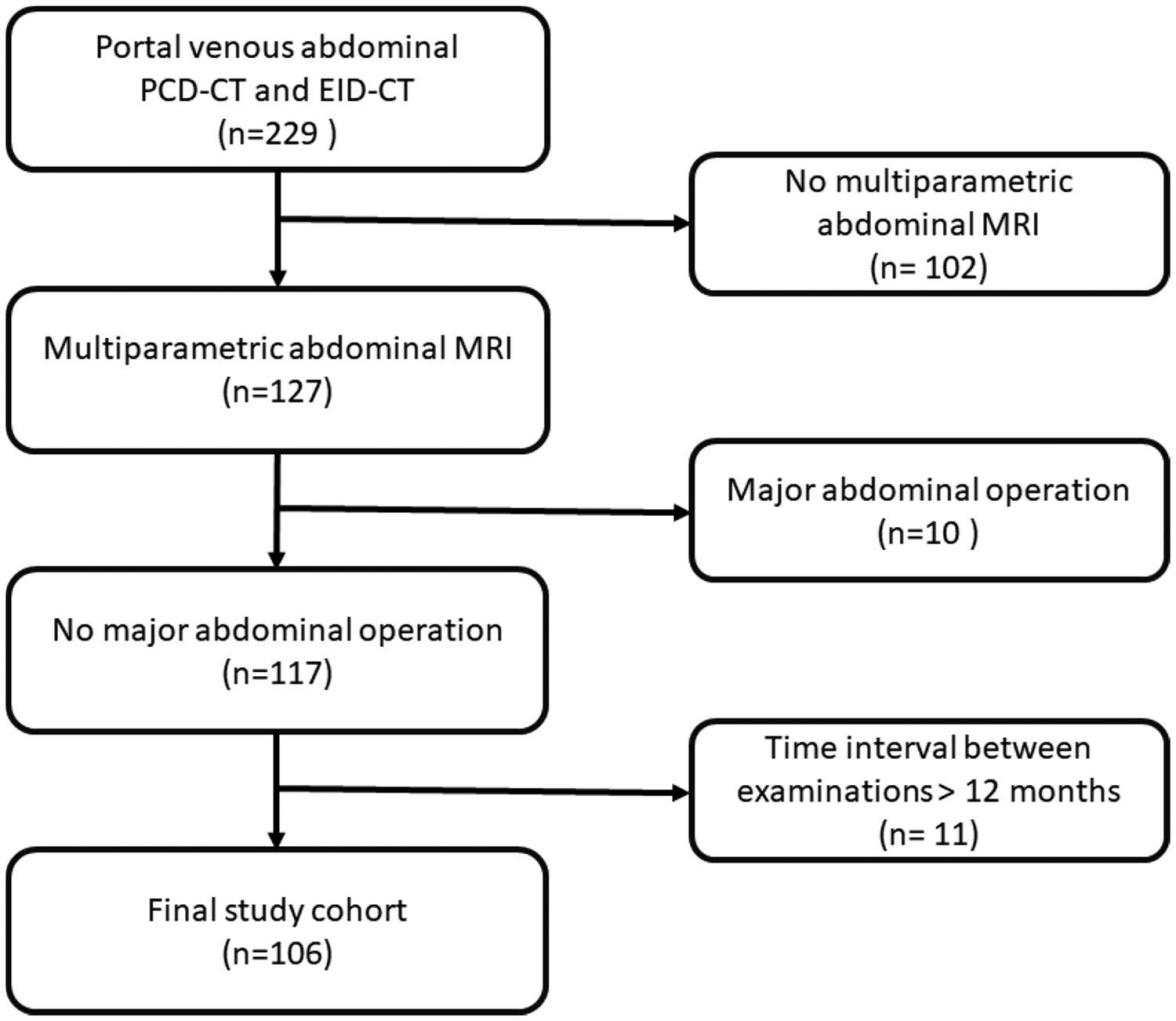
Consort diagram. PCD-CT = photon-counting detector computed tomography; EID-CT = energy-integrating detector computed tomography; MRI = magnetic resonance imaging

Between 02/2022 and 10/2023, 229 patients with oncologic diseases who underwent clinically indicated contrast-enhanced PCD-CT and EID-CT of the abdomen were included in this study. One hundred and two patients had to be excluded because no clinically indicated abdominal MRI was available within 12 months. Additional ten patients had to be excluded due to major abdominal surgery with severe modification of the abdominal anatomy between the EID-CT and PCD-CT scan. Last, 11 more patients had to be excluded because the time interval between the CT and MRI examinations was longer than 12 months (mean time interval between CT and MR imaging was 6.3 ± 2.8 months) resulting in 106 patients included for final analysis (see Fig. 2).

Mean patient age was 62.7 ± 12.6 years, 42.5% (*n* = 45) were male, and the mean BMI was 25.8 ± 4.8 kg/m^2^. In total, 46 patients were diagnosed with a PCL on MRI with a mean size of 8.5 ± 7.5 mm (range 2–45 mm). No worrisome or high-risk features were detected. Further patient characteristics are presented in Table 1.

**Table 1:** Patient characteristics and radiation dose

Characteristics		
Age (years)		62.7 ± 12.6
Sex		45 male (42.5%)
BMI (kg/m^2^)		25.8 ± 4.77
Pancreatic cystic lesions	All lesions	46 (43.4%) in 106 patients
	Lesions ≥ 5 mm	27 (25.5%)
	Lesions ≥ 10 mm	14 (13.2%)
	Mean size (mm)	8.4 ± 7.4
	Range (mm)	2—45
CTDIvol (mGy)	PCD-CT	7.13 ± 2.61
	EID-CT	8.68 ± 3.53

*PCD-CT* Photon-counting detector computed tomography, *EID-CT* Energy-integrating detector computed tomography, *BMI* body mass index, CTDIvol volume CT dose index

### Diagnostic accuracy for the detection of PCL

The diagnostic accuracy for the detection of PCLs (*n* = 46) in all included patients was significantly higher for PCD-CT vs. EID-CT (AUC 0.81 vs. 0.74; *p* = 0.016, respectively). Sensitivity was 76.8% vs 59.4% for PCD-CT vs. EID-CT, and specificity 84.4% vs. 88.3%, respectively. No worrisome or high-risk features of the PCL were identified by the readers.

**Fig. 3:**
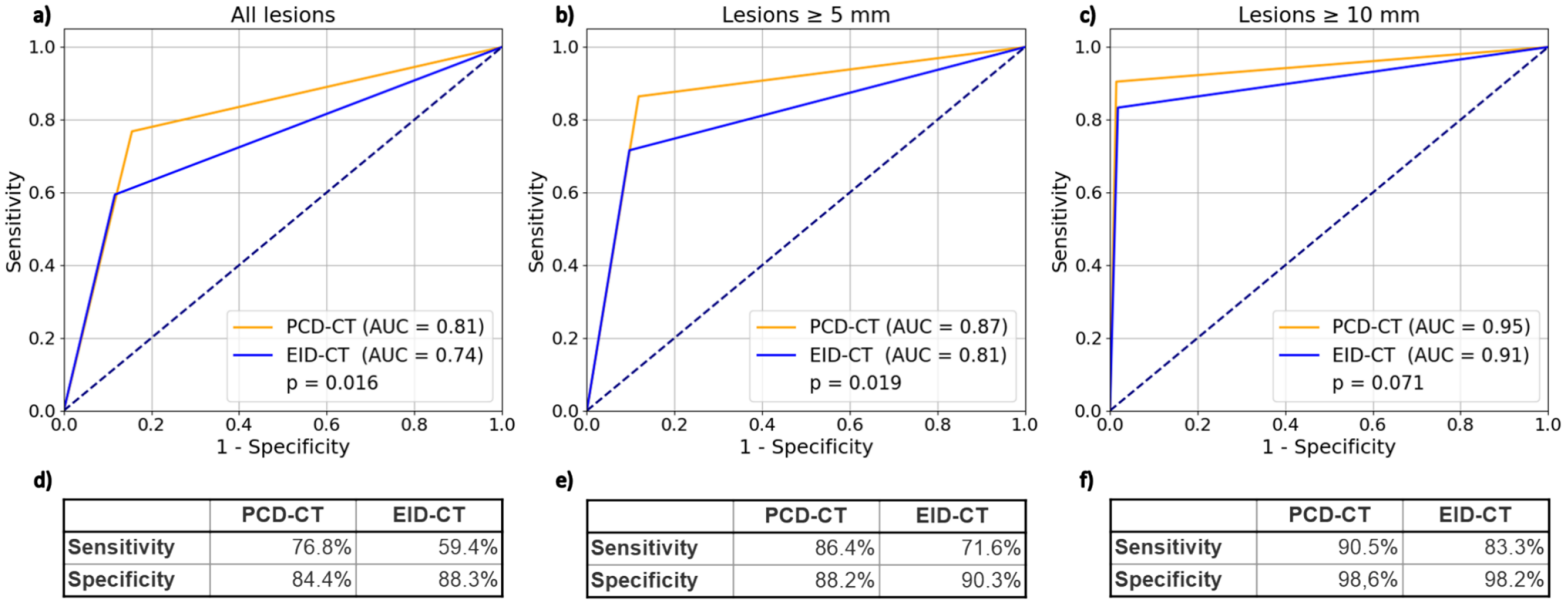
Diagnostic accuracy for PCL detection. ROC AUC for all lesions (**a**), lesions with a threshold size ≥ 5 mm (**b**) and a threshold size ≥ 10 mm (**c**). **d**–**f** depict the sensitivity and specificity. PCD-CT = photon-counting detector computed tomography; EID-CT = energy-integrating detector computed tomography; HU = Hounsfield units; receiver operating characteristic = ROC; area under the curve = AUC

Similar results were found in a sensitivity analysis for PCLs with a threshold diameter of ≥ 5 mm (*n* = 27; AUC 0.87 vs. 0.81; *p* = 0.018, sensitivity 86.4% vs. 71.6% for PCD-CT vs. EID-CT; specificity 88.2% vs. 90.3%, respectively). For lesions with a threshold diameter of ≥ 10 mm, a similar trend was observed without reaching statistical significance (*n* = 14; AUC for PCD-CT vs. EID-CT: 0.95 vs. 0.91; *p* = 0.071, sensitivity 90.5% vs. 83.3% for PCD-CT vs. EID-CT; specificity 98.6% vs. 98.2%, respectively) (Fig. 3). A detailed summary of all results is provided in Table 2.

**Table 2:** Detection performance of PCD-CT and EID-CT for the detection of PCLs

		Pooled results		Reader 1		Reader 2		Reader 3	
PCL size		PCD-CT (%)	EID-CT (%)	PCD-CT (%)	EID-CT (%)	PCD-CT (%)	EID-CT (%)	PCD-CT (%)	EID-CT (%)
All (*n* = 46)	Sensitivity	76.8	59.4	82.6	60.9	82.6	65.2	65.2	52.2
	Specificity	84.4	88.3	88.3	95.0	83.3	93.3	81.7	64.0
≥ 5 mm (*n* = 27)	Sensitivity	86.4	71.6	81.5	70.1	100.0	81.5	77.8	63.0
	Specificity	88.2	90.3	81.0	91.1	88.6	94.9	95.0	84.8
≥ 10 mm (*n* = 14)	Sensitivity	90.5	83.3	78.6	78.6	100.0	92.9	92.9	78.6
	Specificity	98.6	98.2	98.9	98.9	96.7	97.8	100.0	97.8

*PCD-CT* photon-counting detector computed tomography, *EID-CT* Energy-integrating detector computed tomography, *PCL* pancreatic cystic lesion

### Size measurements for detected PCLs

The mean deviation in size measurements for PCLs, as determined by MRI, was 0.6 mm when compared with PCD-CT and 2.3 mm when compared with EID-CT. Post hoc analyses showed no significant differences between MRI-based measurements and those obtained using PCD-CT (*p* = 0.868) or EID-CT (*p* = 0.598). Further detailed comparisons and statistical summaries are presented in Table 3.

**Table 3:** Size measurements for MRI-verified PCLs detected in PCD-CT and EID-CT

	MRI	PCD-CT		EID-CT	
PCL size	Mean size [± *σ*]	Mean size [± *σ*]	Mean difference from MRI [*p*-value]	Mean difference from MRI [± *σ*]	Mean difference from MRI [*p*-value]
All (*n* = 73)	10.7 ± 7.9 mm	10.1 ± 6.6 mm	0.6 mm [0.868]	9.6 ± 7.6 mm	1.2 mm [0.598]
≥ 5 mm (*n* = 56)	12.9 ± 7.8 mm	11.7 ± 8.0 mm	1.1 mm [0.702]	11.1 ± 8.0 mm	1.8 mm [0.437]
≥ 10 mm (*n* = 35)	16.5 ± 7.9 mm	14.6 ± 6.9 mm	1.9 mm [0.573]	13.8 ± 8.9 mm	2.7 mm [0.344]

*EID-CT* Energy-integrating detector computed tomography, *PCD-CT* Photon-counting detector computed tomography, *PCL* pancreatic cystic lesion

### Qualitative image analysis

**Fig. 4:**
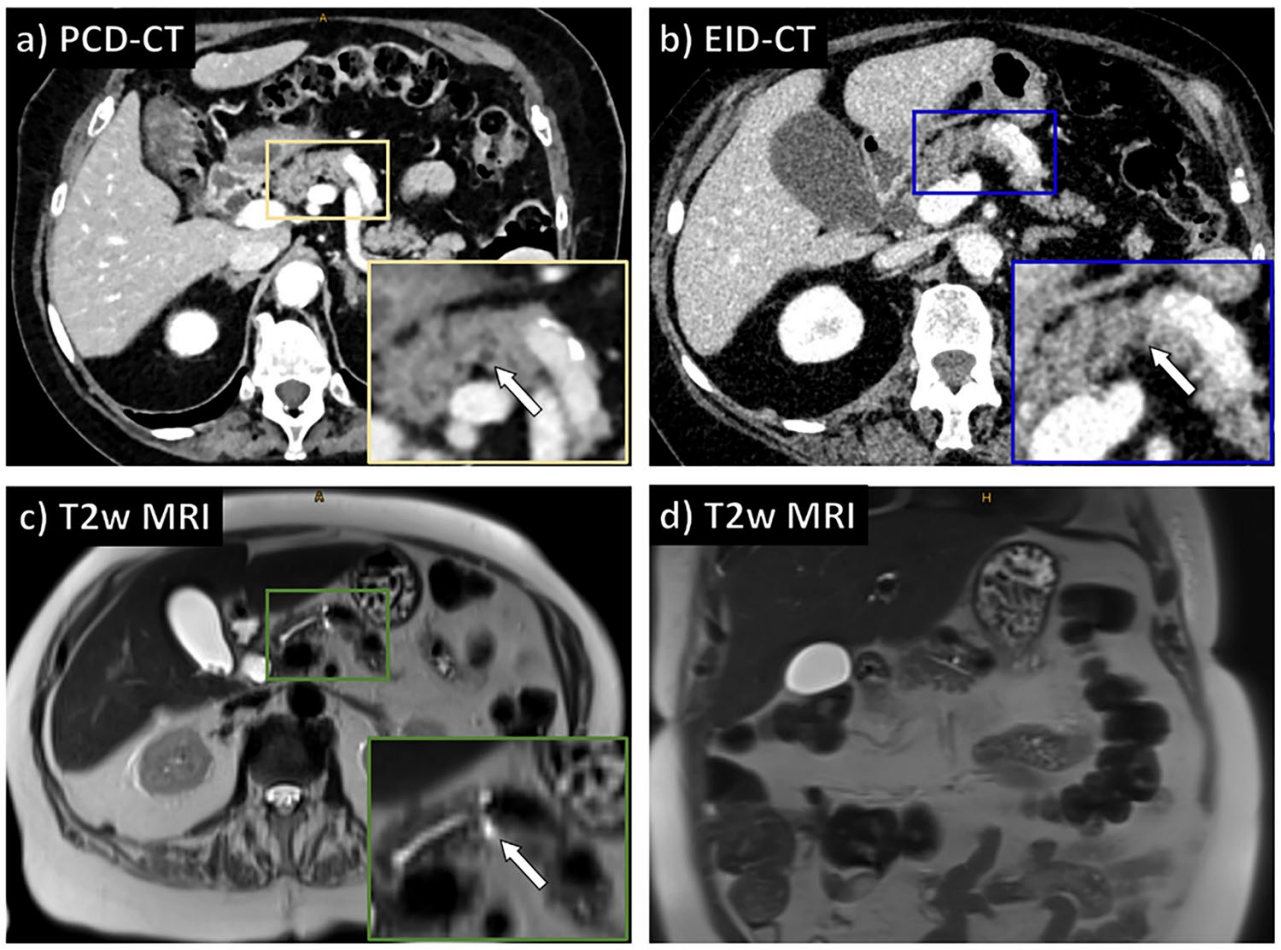
Image quality of PCD-CT vs. EID-CT for the detection of PCL. Images of an 83-year-old female patient who underwent repetitive MRI and CT imaging of the abdomen in the context of an osseous metastasized breast carcinoma. Axial PCD-CT reconstructions (**a**) show a small PCL (3 × 4 mm; white arrow) that is difficult to delineate in the EID-CT scan (**b,** white arrow). MRI references images clearly depict the PCL in axial (**c**) and coronal (**d**) T2-weighted images (white arrow). PCD-CT = photon-counting detector computed tomography; EID-CT = energy-integrating detector computed tomography

**Fig. 5:**
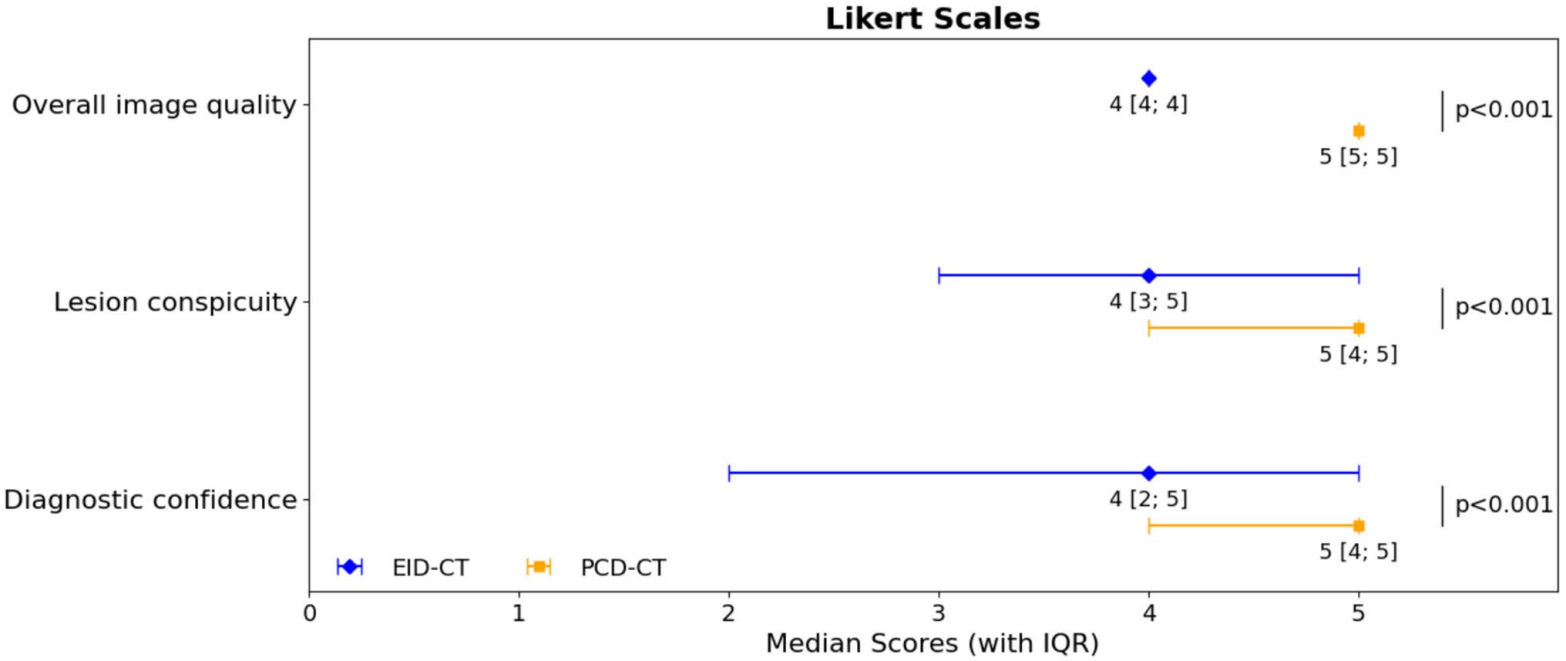
Results of the qualitative image analysis. Pooled qualitative reading scores revealed a significantly superior image quality, lesion conspicuity and diagnostic confidence for PCD-CT vs. EID-CT. PCD-CT = photon-counting detector computed tomography; EID-CT = energy-integrating detector computed tomography; IQR = interquartile range

Overall image quality was high with significantly superior ratings for PCD-CT compared to EID-CT (5 [5; 5] vs. 4 [4; 4], *p* < 0.001). Similar results were found for lesion conspicuity (5 [4; 5] vs. 4 [3; 5], *p* < 0.001) and diagnostic confidence (5 [4; 5] vs. 4 [2; 5]; *p* < 0.001). An image example is provided in Fig. 4, and a summary of all reading results can be found in Fig. 5.

### Quantitative image analysis

#### Quantitative assessment of lesion conspicuity

**Fig. 6:**
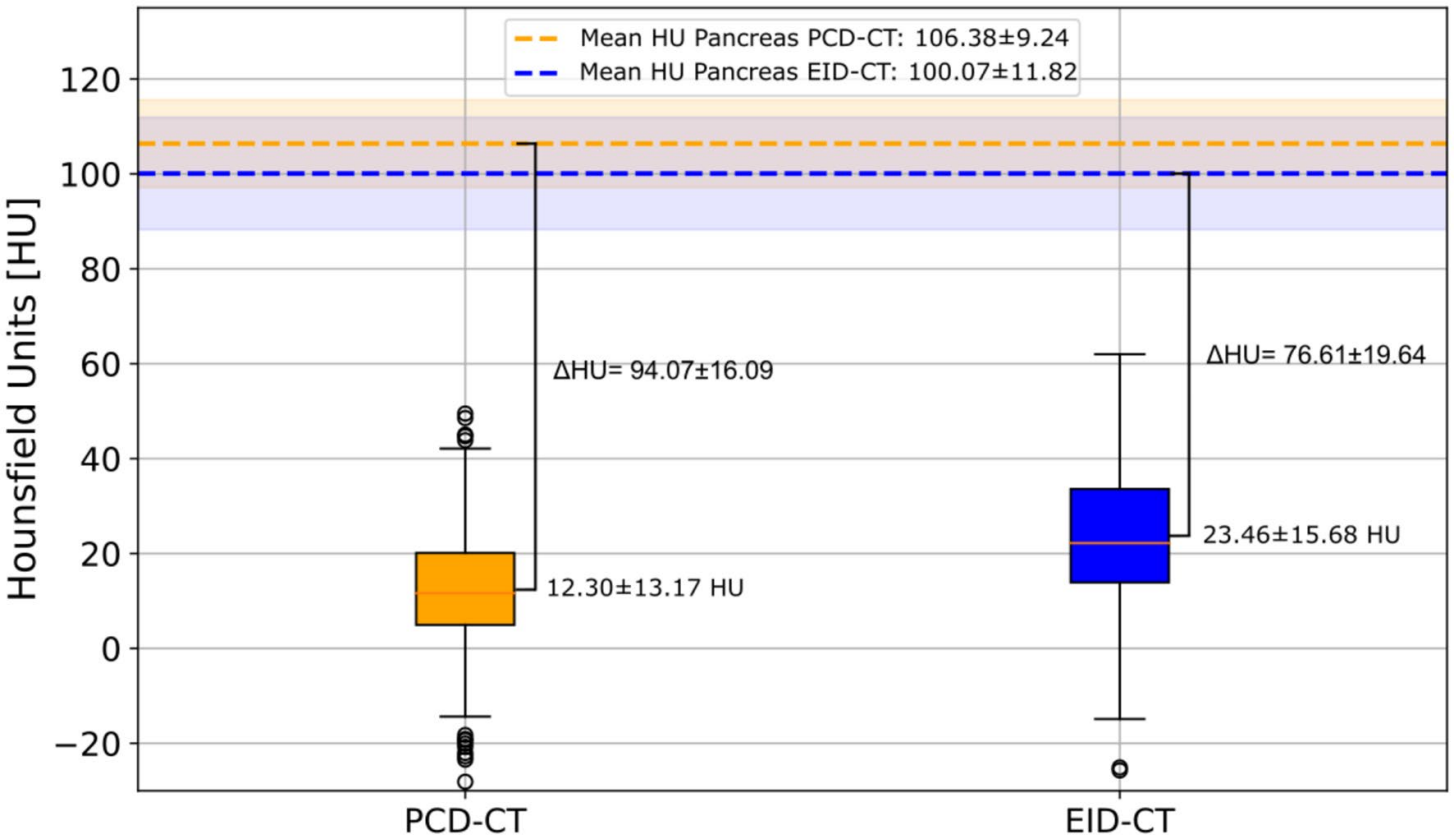
Assessment of quantitative lesion conspicuity. Box plots indicate the significantly different mean HU density in the PCL for PCD-CT (yellow) vs. EID-CT (blue) with *p* < 0.001. The dashed horizontal lines represent the mean HU density with standard deviations of visually normal pancreatic parenchyma for PCD-CT (yellow) and EID-CT (blue). PCD-CT shows a significantly higher HU delta between visually normal pancreatic parenchyma and PLCs compared to EID-CT (*p* < 0.001). PCD-CT = photon-counting detector computed tomography; EID-CT = energy-integrating detector computed tomography; PCL = pancreatic cystic lesion; HU = Hounsfield units

Quantitative analysis of PCL conspicuity revealed superior results for PCD-CT compared to EID-CT with a significantly higher density difference between visually normal pancreatic tissue and the PCL (94.1 ± 16.1 HU vs. 76.6 ± 19.6 HU; *p* < 0.001). This effect was due to significantly lower HU values within PCLs in PCD-CT compared to EID-CT (12.3 ± 13.2 HU vs. 23.5 ± 15.7 HU; *p* < 0.001), whereas no significant HU difference was found for visually normal pancreatic parenchyma (106.4 ± 9.2 HU vs. 100.1 ± 11.8 HU; *p* = 0.175) (Fig. 6).

#### Evaluation of CT signal homogeneity

CV analysis revealed a significantly more homogenous image signal for PCD-CT examinations compared to the traditional EID-CT with a mean CV of 0.19 ± 0.16 vs. 0.24 ± 0.22; *p* = 0.002, respectively.

### Radiation dose

Radiation dose was significantly lower for PCD-CT (7.13 ± 2.61 mGy) compared to EID-CT (8.68 ± 3.53 mGy; *p* < 0.001).

## Discussion

In this study, we investigated the diagnostic accuracy of PCD-CT for the detection of PCL and found a significantly improved performance compared to conventional EID-CT in routine abdominal imaging studies. In addition, PCD-CT provided superior subjective and objective image quality at significantly lower radiation dose.

These findings are of clinical importance, as PCLs are a common incidental finding in abdominal imaging studies and reliable and accurate detection is of great importance as a substantial number can be considered as premalignant or malignant lesions that require further workup or immediate treatment as they have been reported to be associated with an increased rate of mortality [4, 12]. Our results suggest that PCD-CT could help to identify significantly more individuals with PCLs in routine clinical care that would otherwise go unnoticed. We attribute this to two features of the novel PCD technology: First, the PCD provides a higher spatial resolution due to the different detector architecture contributing to a superior lesion conspicuity and edge sharpness. Second, the possibility to count and measure the energy of every photon at the detector allows for improved image contrast by a relative overweighting of low-energy photons compared to EID-CT systems and by reducing (electronic) noise [13, 14]. These theoretical considerations are supported by our results, which indicate a significantly higher overall image quality and lesion conspicuity of PCD-CT compared to conventional EID-CT systems resulting in a higher diagnostic confidence of the radiologist reading the scans. One can expect an even more pronounced improvement of PCL detection in PCD-CT in comparison with older EID-CT systems. Noteworthy, the data indicate that the significantly improved accuracy for PCD-CT for PCLs is mainly attributed to the improved sensitivity, while the specificity remains similar to EID-CT. Thus, the improved image quality allows for better lesion detection, but at the expense of a possible confusion of cystic lesions with small fatty lesions. The comparison of size measurements across modalities demonstrated no significant differences between MRI, PCD-CT, and EID-CT. Despite these similarities, the sensitivity of PCD-CT for PCL detection remains a key advantage, particularly in detecting smaller lesions.

The results of the improved subjective image quality analysis and accuracy are corroborated by the quantitative image assessments. While HU measurements in visually normal pancreatic parenchyma revealed similar results for PCD-CT and EID-CT scans, HU values in PCLs were significantly lower in PCD-CT compared to EID-CT examinations resulting in a higher parenchyma-to-lesion contrast. We attribute the HU difference within the cysts between the systems to improved material decompensation through better weighting of the low-energy photons in the PCD-CT, whereby the photons with high image information contribute significantly to better soft tissue contrast. In addition, the coefficient of variation analysis indicated that the acquired PCD signal is significantly more homogeneous compared to the EID signal, which further supports lesion conspicuity. These results are in line with previous studies investigating cystic lesions in the kidneys, which demonstrated a more accurate differentiation of lesion composition for PCD-CT compared to EID-CT [15, 16]. Moreover, EID-CT systems require special dual-energy capabilities for advanced post processing and material decomposition, which comes at the cost of higher complexity and potentially more radiation dose, depending on the respective technology. In contrast, in the presented study, we found that PCD-CT offered multispectral data in every scan at significantly lower radiation dose compared to the latest state-of-the-art dual-energy EID-CT system. Whether these multispectral data can be used for further characterization and detection of potential malignant transformation of PCLs or differentiate between different PCL entities was beyond the scope of the current study and should be subject of further focused research.

There are limitations that need to be considered. First, this was a cross-sectional imaging study with no long-term follow-up and/or histopathological correlation of the detected PCLs. While histopathology is considered the gold standard for definitive diagnosis, it was unavailable in this study because no pancreatic resections or autopsies were performed in the final study group. Instead, we relied on MRI as the clinical reference standard, which is widely accepted for the characterization of pancreatic cystic lesions in clinical practice [7]. Further, only the detection of PCLs was investigated, and no further lesion characterization including the multispectral PCD-CT imaging information was performed. Finally, only the institutional standard imaging protocols for PCD-CT and EID-CT were evaluated. Optimizing monoenergetic and iterative reconstruction settings might help to further improve detection of PCLs. As malignant and premalignant lesions were resected shortly after the initial imaging studies, it was not possible to acquire a follow-up CT scan of these lesions. Hence, the PCLs included in this study were most likely either pseudocysts or side-branch IPMNs; however, due to the small lesion size and missing histopathology, no final diagnosis could be made. Thus, the treatment-decisive diagnostic value of PCD-CT in differentiating side branch and main duct IPMN remains unclear; therefore, an important focus for subsequent studies.

PCD-CT provided significantly higher diagnostic accuracy and superior image quality for the detection of PCLs compared to conventional EID-CT at lower radiation dose. This may help to better identify patients with PCL in daily routine that would otherwise go unnoticed and initiate personalized workup and risk assessment.

## Abbreviations

AUC: Area under the curve
CTDI: CT dose index
CV: Coefficient of variation
EID-CT: Energy-integrating detector CT
PCD-CT: Photon-counting CT
PCL: Pancreatic cystic lesion
ROC: Receiver operating characteristic
ROI: Region of interest
